# Physical health and symptoms of relative energy deficiency in female fitness athletes

**DOI:** 10.1111/sms.13568

**Published:** 2019-10-20

**Authors:** Therese Fostervold Mathisen, Josefine Heia, Marius Raustøl, Mari Sandeggen, Ingrid Fjellestad, Jorunn Sundgot‐Borgen

**Affiliations:** ^1^ Department of Sports Medicine Norwegian School of Sport Sciences Oslo Norway; ^2^ Department of Sport Performance Norwegian School of Sport Sciences Oslo Norway; ^3^ School of Clinical and Applied Sciences Leeds Beckett University Leeds UK; ^4^ Faculty of Health Sciences Oslo Metropolitan University Oslo Norway

**Keywords:** amenorrhea, body composition, eating disorders, fitness physique, low energy availability, relative energy deficiency in sport, resting metabolic rate

## Abstract

**Introduction:**

Competing in aesthetic sports increases the risk of low energy availability and associated health impairments. Fitness physique sport is a popular, but understudied aesthetic sport. We evaluated health and symptoms of relative energy deficiency in sport (RED‐s) in female fitness athletes (FA) and female references (FR) during a competitive season.

**Methods:**

Totally, 25 FA and 26 FR, mean (SD) age of 28.9 (5.7), were included. Assessments were at baseline (T1), 2‐weeks pre‐competition (T2), and 1‐month post‐competition (T3), by dual‐energy x‐ray absorptiometry scan, indirect calorimetry, diet registration, The Low Energy Availability in Females Questionnaire, The Beck Depression Inventory, and Eating Disorder Examination Questionnaire (EDE‐Q).

**Results:**

A history of eating disorders was reported by 35% FA and 12% FR. There were no between‐group differences at T1, besides less mean (99% CI) fat mass (FM) of 3.1 kg (−0.4, 6.5) in FA (*P* = .02). At T2, FA had lower BW of 6.7 kg (−12.0, −1.3), fat mass of −9.0 kg (−12.5, −5.5), and resting heart rate of −8.0 beats per minute (−14.5, −1.5) compared to FR (*P* ≤ .006). FA reduced resting metabolic rate by −191 kcal (−11, −371) and increased symptoms of gastrointestinal dysfunction (GD) by 1.4 points (0.3, 2.5) and prevalence of amenorrhea from 8% to 24%, (*P* < .003). At T3, there was a between‐group difference in fat mass, and a high number of FA with amenorrhea and GD.

**Conclusion:**

Manifestation of symptoms of RED‐s, some with persistence one‐month post‐competition, raises concern for the health of FA and those complying with the fit body ideal.

## 1 INTRODUCTION

The fitness physique sport in federations of bodybuilding, with its many sub‐categories (ie, fitness, bikini fitness, wellness fitness, body fitness, fitness physique), is a rather new phenomenon.^1^ This is comparable to bodybuilding by emphasizing a lean body composition, although with less muscle mass, vascularization, and muscle definitions.^1^, ^2^, ^3^ With these new criteria, the sport now specifically appeals to females, consolidating with the modern, athletic toned female body ideal.^4^ By emphasizing regular exercise and eating well, the sport is also promoted as a healthy lifestyle, serving as an improved ideal to the former thinness inspiration.^2^, ^4^ In this sport, athletes are exclusively rated subjectively by judges on their aesthetic appearance, emphasizing a lean, toned figure,^1^, ^2^, ^3^ which has been reported as a risk factor for extreme dieting and disordered eating.^3^, ^4^, ^5^

The use of unhealthy and harmful methods for body weight (BW) regulation is common in organized sports characterized as weight‐sensitive or organized according to weight classes.^5^ These methods may range from fasting, skipping meals, and use of different dehydration techniques, to the use of purging methods like self‐induced vomiting, laxatives, diuretics, and excessive exercise.^5^, ^6^ Such weight regulation practices strongly associate with impaired physical and mental health, and athletic performance.^7^ Low energy availability (LEA) during extended periods among athletes might bring up symptoms of relative energy deficiency in sport (RED‐s, defined as “impaired physiological functioning caused by relative energy deficiency, and includes but is not limited to impairments of metabolic rate, menstrual function, bone health, immunity, protein synthesis, and cardiovascular health”).^7^ Females are prone to such impairments, which specifically raise concern to the potential detrimental effect on bone mineral density (BMD), and hormonal disturbances affecting metabolism and fertility.^7^, ^8^ Hence, recommendations for optimal regulation of BW and body composition have been suggested, in order to minimize the risk of negative health consequences.^5^, ^6^, ^9^, ^10^ These guidelines specifically emphasize frequent and regular meal intake, high protein intake, adequate carbohydrate intake, saturating diets (eg, high in dietary fiber), slow rate of weight reduction, and a moderate energy deficit.^5^, ^6^, ^9^, ^10^

Bodybuilders and fitness athletes (FA) are found to comply with these suggested "best methods".^3^, ^11^, ^12^, ^13^ These athletes keep track of their total energy and macronutrient intake, eat fibrous foods, follow a high‐protein diet, and diet for several weeks to allow for a slow rate of BW loss.^3^, ^11^, ^12^, ^13^, ^14^, ^15^, ^16^, ^17^ The athletes also prepare meals for several days ahead, eat frequently during the day, and make sure to adjust meal intake according to the exercise sessions.^12^, ^13^, ^14^, ^15^ Additionally, they emphasize resistance exercise,^13^, ^14^, ^15^, ^16^, ^17^ which specifically may be favorable in reducing the detrimental effect of dieting and low BW on lean body mass and BMD.^5^, ^13^, ^14^, ^15^, ^16^, ^17^, ^18^, ^19^ However, FA are understudied with regard to physical and mental health variables, like the ones identified as symptoms of RED‐s.^1^, ^3^, ^7^ Previous studies of athletes in bodybuilding sports reveal issues with binge eating, and report on a high prevalence of previous eating disorders, specifically among females.^3^ Recent findings in groups of fitness athletes are indications of a continued, strict dieting behavior beyond the competitive season, not to enhance performance, but to comply with the modern lean and fit body ideal.^3^, ^4^ Hence, there are reasons for concern for the health of these athletes. At the time, only four case studies^14^, ^15^, ^16^, ^17^ and four cohort publications^18^, ^19^, ^20^, ^21^ report on health outcomes in females related to dieting for fitness sport participation. Among the publications with post‐competition measures,^14^, ^17^, ^20^, ^21^ changes in BW and composition were reported to be temporary, with return to or above baseline^14^, ^17^, ^20^ or normal, healthy levels^21^ within 1‐4 months post‐dieting. Unfortunately, shortages in these studies are either lack of reports on BMD,^14^, ^16^, ^17^, ^21^ lack of psychometric outcomes,^14^, ^15^, ^21^ lack of post‐competition measures,^15^, ^16^ or lack of comparisons to a healthy reference group not participating in this sport.^14^, ^15^, ^16^, ^17^, ^20^, ^21^ Furthermore, site‐specific BMD, such as spine BMD and proximal femur BMD, is more prone to the negative effect from low BW or dieting, while total BMD is less likely to reflect early changes.^24^ Only one of the previous studies reported on site‐specific BMD, concluding with no negative effect, despite reporting a seemingly overall low BMD.^15^ Hence, there is a lack of a comprehensive evaluation of health and symptoms of RED‐s before, during and after a period of contest preparation for this specific group of sport athletes.

There has been a tremendous increase in interest of fitness sports in which athletes exclusively are rated by their aesthetic appearance.^1^, ^2^ With the lack of knowledge on how this specific lifestyle might affect young athletes, we aimed to study the physical and mental health in female FA during a competitive season. A reference group of recreationally active and non‐competing females (female references, FR) was included to provide information on whether findings are sport‐specific, or general findings in a modern sample of young females, and to control for the effect of time. Hence, this explorative study evaluates the health and symptoms associated with RED‐s (ie, low energy availability, menstrual disturbances, gastrointestinal dysfunction, low resting metabolic rate, bradycardia, psychological deteriorations, reduced bone mass, and reduced muscle and fat mass) in female FA competing in fitness physique sports, and compares the outcomes to FR. We hypothesized that:

1. At baseline (T1), the FA group has more lean body mass (LBM), less fat mass, comparable BMD, and more symptoms of ED and depression, compared to the FR group,
2. At time of competition (T2), the FA group reveal more symptoms of RED‐s than the FR group,
3. For the FA group, the symptoms of RED‐s at one‐month post‐competition (T3) are similar to T1, and not different from the FR group
4. Within the FA group, more successful FA (ie, those placing among top five) have more LBM, less fat mass, and more symptoms of RED‐s compared to less successful ones (ie, those placing below top five).

## 2 MATERIALS AND METHODS

This cohort study was conducted at the Norwegian School of Sport Sciences (NSSS) during 2017. We performed no power calculation, due to the explorative approach of this study.

### 2.1 Ethics

The study was approved by the Norwegian Regional Committee for Medical and Health Research Ethics (ID:2016/1718) and registered in Clinical Trials (ID: NCT03007459). Included participants signed an informed consent, first distributed by email and returned during the first physical screening procedure.

### 2.2 Recruitment and participants

We recruited female FA planning to compete in the fitness categories in the upcoming Norwegian national competitions during spring and fall season 2017, and FR, all aged 18 to 40 years. A webpage was created with recruitment information, targeting both the FA and the FR. The webpage was distributed in social media and emailed to all coaches officially listed by the Norwegian Federation of Bodybuilding and Fitness (http://www.nkf-ifbb.no). Additional inclusion criteria for the FR were a body mass index (BMI, kg m^−2^) between 17.5 and 30 and being regularly physically active (ie, being recreational active in any kind of sport/physical activity for at least 2 sessions per week in the last year). All responding FA in the defined age range, planning to compete in the upcoming season (ie, confirming such plans, and initiating a diet for such attendance after baseline measures), and willing to participate in this cohort study, were included. Exclusion criteria for FR were being or planning to become pregnant, former experience from competitive fitness‐ and bodybuilding sports, or plans for such attendance, as well as being a personal trainer or instructor in the fitness industry. All interested participants were interviewed according to the inclusion and exclusion criteria and informed about the project by telephone.

**Figure 1:**
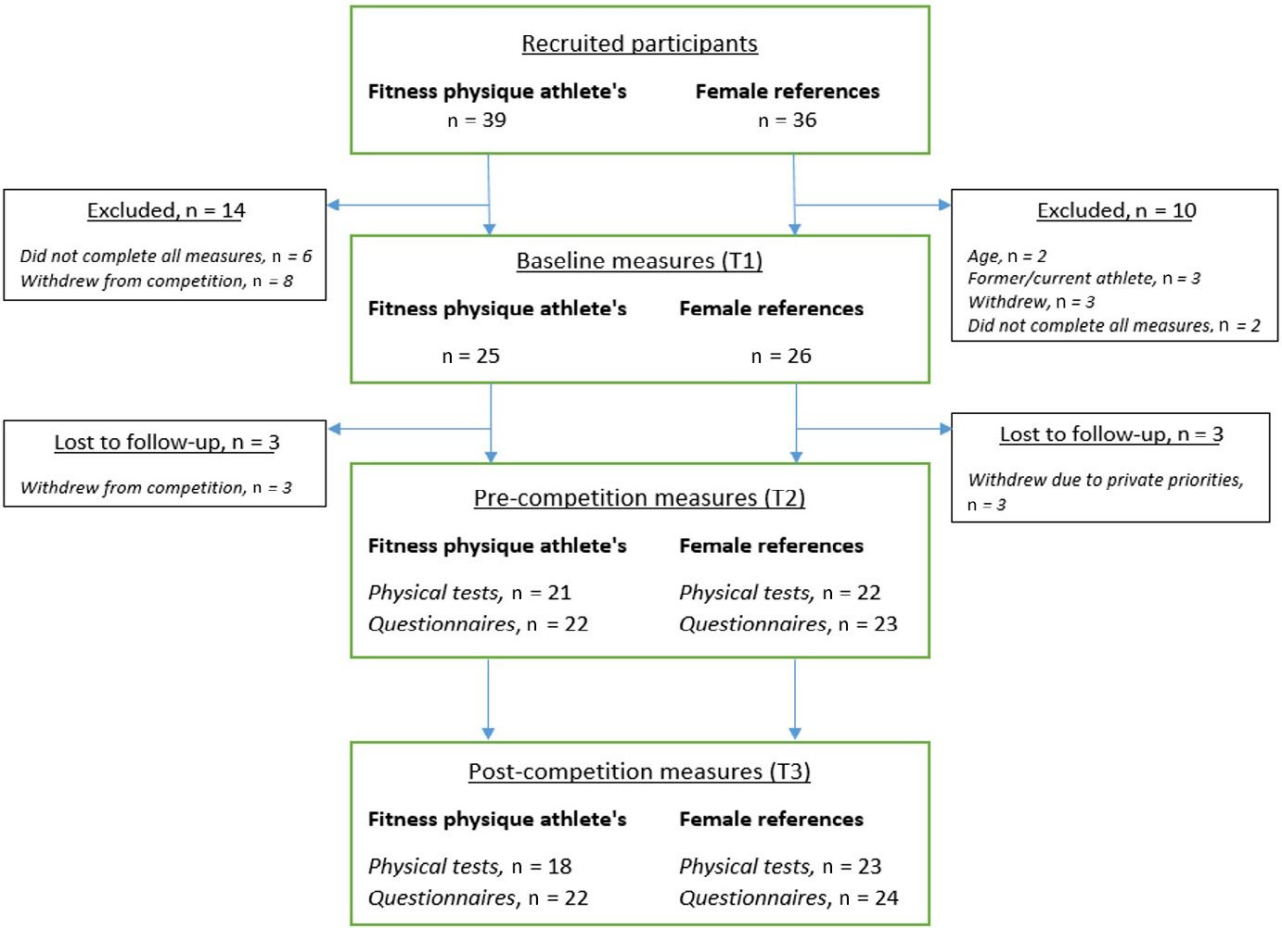
Overview of numbers of participants recruited and included in the two groups during the three evaluation periods. Only participants with complete baseline measures were included

In total, 39 FA and 36 FR responded to recruitment. Only participants with full baseline screening were included in this study, hence comprising 25 FA and 26 FR (Figure 1). Among the 25 FA, nine had competed previously (range: 1‐7 competitions), and totally 21 (84%) were bikini fitness athletes, three (12%) were body fitness athletes, and one (4%) was an athletic fitness athlete (Nordic competition category, including physique evaluation, strength performance elements, and obstacle course). All were competing in national championships in Norway during 2017 including two FA who made the podium in international championships. To study any difference between successful athletes (placing among top five in individual height classes) and less successful athletes (placing lower than among top five in individual height classes) in relevant outcomes at T2, we did separate subgroup analysis.

### 2.3 Design

We provided no intervention, and as such; FR followed their own preferred diet and exercise regime, while FA followed their personal diet plan and exercise routine. All participants were asked to meet for physical assessment at the NSSS before the dieting procedure for the FA was initiated (ie, 3‐4 months before competition depending on the length of the kcal‐reduced dieting period for the individual athletes), to provide baseline data (T1). The same test procedure was conducted 2 weeks prior to the first fitness competition (T2), and then finally one‐month post‐competition (ie, 1 month after the last competition for the individual fitness competitor) (T3). Totally, 13 athletes competed in one competition only, seven athletes competed in two competitions separated by 14 days, and two athletes competed in three competitions separated by a total of 21‐56 days.

### 2.4 Methods and questionnaires

Participants were required to conduct a 12‐hour overnight fast and to abstain from intense exercise 24 hours prior to laboratory assessments. They were asked to arrive at NSSS by passive transportation and meet in the laboratory between 07.30 and 10.00 am at the three physical assessment times (T1‐T3). The test battery included electronic questionnaires at the three screening time points with general demographic questions, including self‐reported history of ED and training experience, and a selection of psychometric questionnaires. Relevant validated questionnaires for the current publication are the Eating Disorder Examination Questionnaire (EDE‐Q),^25^ the Low Energy Availability in Females Questionnaire (LEAF‐Q),^26^ and the Beck Depression Questionnaire (BDI‐1a).^27^ All participants also completed four days of weighed diet registration at each of the test occasions. The physical tests included indirect calorimetry to measure resting metabolic rate (RMR), a measure of resting heart rate, and a dual‐energy x‐ray absorptiometry (DXA) for measure of body composition.

#### 2.4.1 The EDE‐Q

All participants completed the EDE‐Q (Cronbach's α = .93), which comprises 18 items scored 0‐6 to measure the presence (12 items) and the frequency (6 items) of core ED‐characteristics.^25^ A global cutoff score of 2.5 has proved valid in identifying the probability of an ED among Norwegian female adults, with higher scores indicating increased severity of symptoms of ED.^28^

#### 2.4.2 LEAF‐Q

The LEAF‐Q was developed to screen for LEA in female athletes and measure occurrence of injuries, gastrointestinal dysfunction (GD), and menstrual irregularities (MI) related to LEA.^26^ The sensitivity and specificity have proven optimal to classify current energy availability, reproductive function, and/or bone health in endurance athletes and dancers.^26^ Suggested cutoffs for GD, MI, and for the total LEAF‐Q score are ≥2, ≥4, and ≥8, respectively, with higher scoring indicating more severe clinical condition.^26^ Among those with no hormonal contraceptives (HC) (FA, n = 10 [40%], FR, n = 8 [31%]), the reported numbers of menstrual cycles during the last year were used to categorize oligomenorrhea (≤9 cycles), while absence of at least three consecutive menstrual cycles was used to categorize amenorrhea.

#### 2.4.3 BDI‐1a

The BDI‐Ia measures current (past two weeks) self‐reported symptoms of depression.^27^ It consists of 21 items scored on a 4‐point Likert scale ranging from 0 (not at all) to 3 (extreme). Total score range is 0‐63, and a cutoff score of ≥21 is recommended for use to detect a clinically significant episode of major depression.^27^ In the current total sample (FA and FR), Cronbach's α = .83, based on T1‐scorings. By removing one item related to recent weight loss, the Cronbach's α = .86.

#### 2.4.4 Diet and physical activity assessment

During a period of four days (three weekdays and one weekend day), participants weighed and registered all food, beverages, and supplements consumed. Details on the condition of the foods when being weighed were provided (ie, in raw, fried, or cooked condition), and details on specific brand names or versions of the food items (ie, original or light version) were also given. The food diaries were analyzed by using the Norwegian analytical software Diett.no (Brandsar, 06.01.2001), a program with information based on the Norwegian Food Composition Table 2018 (The Norwegian Food Safety Authority and the Department of Nutrition at the University of Oslo). Four athletes reported their diet program at T2 and T3 rather than actual intake, but as fitness athletes are very accurate on their detailed diet, also such reports were included.^3^, ^12^, ^14^, ^15^, ^16^ The results from the weighed dietary registration were used to calculate energy intake per kg lean body mass, and carbohydrate and protein intake per kg BW.

Frequency and type of physical activity were self‐reported, but as no detailed information on intensity or duration was provided, exercise energy expenditure was not calculated. Hence, the proper calculation of energy availability^29^ was not possible.

#### 2.4.5 Resting metabolic rate (RMR)

To evaluate the effect from dieting on metabolism, RMR was measured by indirect calorimetry using a respiratory gas analyzer (Oxycon Pro, Jaeger, Germany). Ambient conditions were registered and the analyzer was gas and volume calibrated each morning prior to the measurements, according to the recommendations stated in the user manual from the manufacturer (user manual for Oxygen Pro, Jaeger, Germany). Six participants were measured each day. Gas exchange and ventilatory variables were measured continuously using the breath‐by‐breath method and by following the suggested best practice.^30^ Participants were instructed to rest for 10 minutes, wearing a two‐way breathing mask covering their nose and mouth (2700 series; Hans Rudolph, Inc). Thereafter, the measurement period started by connecting the mask to the gas analyzer, and data collection continued for a total of 20 minutes. All data output were given in 30‐ second intervals and calculated as means per minute. To reduce errors caused by gas remaining in the tubes, the data from the first 30 seconds were erased from the analysis. A valid RMR was defined according to the current recommendation emphasizing the importance of a steady state, being defined as 5‐ minute periods with less than 10% CV for VO_2_ and VCO_2_.^30^ A ratio between the measured RMR (RMR_m_) and the theoretically calculated RMR (RMR_c_) by Cunningham formula,^31^ RMRm/RMRc_,_ below 0.9, is commonly used as a threshold for diagnosis of clinically low RMR, indicative of energy deficiency.^32^, ^33^

#### 2.4.6 Resting heart rate (HR)

HR may decrease during starvation or low energy availability, resulting in bradycardia (HR < 60 beats per minute) 34, 35; hence, we included a measure of resting HR. The lowest resting heart rate was noted during the RMR measure, using a SpO_2_ (Welch Allyn Spot Vital Signs LXI Monitor with SureBP, Nellcor Sp0_2_).

#### 2.4.7 DXA

Participants were weighed in their underwear, and their height was measured with a fixed stadiometer (Seca scale, Mod: 8777021094, S/N: 5877248124885, Seca Deutschland, Hamburg, Germany). A DXA (Lunar iDXA, enCORE Software, version 14.10.022; GE Healthcare, Madison, WI) performing a three‐site scan (lumbar area [L2‐L4]; proximal femur [femoral neck, trochanter, and shaft]; whole body) was used to measure body composition (fat mass [kg], percent body fat [%BF], lean body mass [kg], visceral adipose tissue [VAT, gram], and BMD for spine and femur). All measures were done by one of two trained technicians, and all data were analyzed by one technician according to the guidelines.^36^ Due to the lack of reliability evaluation of the specific DXA‐machine and personnel, a previous evaluation of iDXA precision was used to identify the least significant change (LSC) of BMD; being 0.04 g cm^−2^ for spine BMD and 0.03 g cm^−2^ for femur BMD.^37^

#### 2.4.8 Statistics

All analyses were conducted in SPSS version 24. Linear mixed regression models were built to estimate the between‐group differences (FA vs FR) and the within‐group changes (T1 vs the two other measures). This analysis yields relatively unbiased estimates despite drop out, given that data are missing completely at random or missing at random. Standard errors were estimated with the restricted maximum likelihood function. Dependency in the repeated outcome measures was accounted for by including a random intercept factor. The fixed factors were: *Group* (FA, FR), *Time* (T1, T2, T3), and *Group* × *Time*. Differences between the groups were examined with planned comparisons at each time point (least square difference tests). The within‐group analyses included all three measurements in the *Time* factor. Due to the number of tests, differences with *P*‐values ≤ .01 were considered significant. A comparable statistical approach was used for the dichotomous outcome variables, replacing the analysis with a generalized linear model using a binominal distribution and logit link function. Degrees of freedom were computed using Satterthwaite approximation. The outcome data are presented as estimated means including 99% confidence intervals, as given in the analytical software output.

Standardized Hedge's *g* effect sizes for continuous data were calculated as a ratio of the estimated means (extracted from the mixed model) to the observed pooled standard deviations (SD). Values around 0.2, 0.5 and 0.8 were interpreted as weak, medium and strong effect sizes, respectively.^38^

The subgroup analyses of more and less successful FA, and the demographic data presented in Table 1, were analyzed separately with independent t test, Mann‐Whitney U test, or chi‐square analyses as appropriate. A significance level of *P* < .05 was used for the demographic analysis (Table 1).

**Table 1:** Demographic presentation of fitness athletes (FA) and female references (FR) at baseline (T1).

	FA, n = 25	FR, n = 26	*P*‐value, (*effect size*)
Age, years	28.1 (5.5)	29.8 (6.0)	.28
BMI, kg m^−2^	22.5 (2.1)	23.2 (2.9)	.42
Body weight, kg	62.5 (6.9)	64.3 (8.3)	.35
Fat mass, kg	15.1 (4.5)	18.1 (5.7)	**.04,** *(e = −0.53)*
Lean body mass, kg	45.4 (4.3)	44.1 (4.2)	.36
Adult BW difference, kg^a^	15.0 (5.5)	13.0 (12.3)	.43
History of ED, self‐reported, n (%)	9 (34.6%)	3 (12.0%)	.06
Current ED, self‐reported, n (%)	2 (7.7%)	1 (4.0%)	.60
Hormonal contraceptives, n (%)	15 (60.0%)	18 (69.2%)	.49
Experience with regular exercise ≥ 5 y, n (%)^b^	16 (64.0%)	21 (80.8%)	.18
Exercising ≥ 5 times per week current year, n (%)	14 (56.0%)	8 (30.8%)	.07

Note

Values are mean (SD) if not otherwise stated, with effect size of any difference reported with Hedges g.

Abbreviations: BW, body weight; ED, eating disorder; kg, kilogram.

a Numbers are median (IR).

b Regular exercise defined as ≥ 2 sessions per week.

## 3 RESULTS

Attrition analysis revealed no differences between dropouts and completers in FA at T1. Among FR, dropouts consumed less mean (95% CI) energy; 702 (75, 1329) kcal (*P* = .03), compared to completers, but no other differences were identified.

### 3.1 Demographics

Demographic information on participants at T1 is presented in Table 1.

Among those without use of HC in FA, the mean (99% CI) numbers with amenorrhea were 8% (1, 39) at T1, increasing to 24% (8, 53) at T2‐T3 (*P* < .003). The corresponding finding in FR was 12% (2, 41) at T1, with no change by time. Additionally, 8% (2, 26) in FA had oligomenorrhea at T1. In FR, 4% (1, 22) had oligomenorrhea at T1, none at T2, and 8% (2, 25) at T3 (*P* > .2). There were no between‐group differences for menstrual irregularities at any time (*P* > .05).

The average total number of exercise sessions per week during the dieting period for FA (T1‐T2) was 5‐6 sessions in FA group, with 3‐4 sessions of aerobic exercise and 5‐6 sessions of resistance exercise. The corresponding number of sessions in FR was 3‐4 total sessions, 1‐2 aerobic exercise and 1‐2 resistance exercise sessions per week. There was a between‐group difference for the number of resistance exercise sessions, and the tendency of an increased number of total sessions and aerobic sessions in FA during the last 2 months of dieting resulted in between‐group differences (*P* < .005). Additionally, a total of five persons in FA and one person in FR reported doing more than one exercise session per day during the FA dieting period (*P* < .05).

### 3.2 The EDE‐Q

Estimated mean (99% CI) EDE‐Q global score at T1 was 1.4 (0.8, 2.0) in FA and 1.1 (0.5, 1.6) in FR. The estimated mean (99% CI) numbers with EDE‐Q global score above cutoff at T1 were 2 (8%; 1, 39) in FA and 2 (8%; 0, 38) in FR. There were no within‐group changes by time or between‐group differences for EDE‐q results.

### 3.3 LEAF‐Q

**Figure 2:**
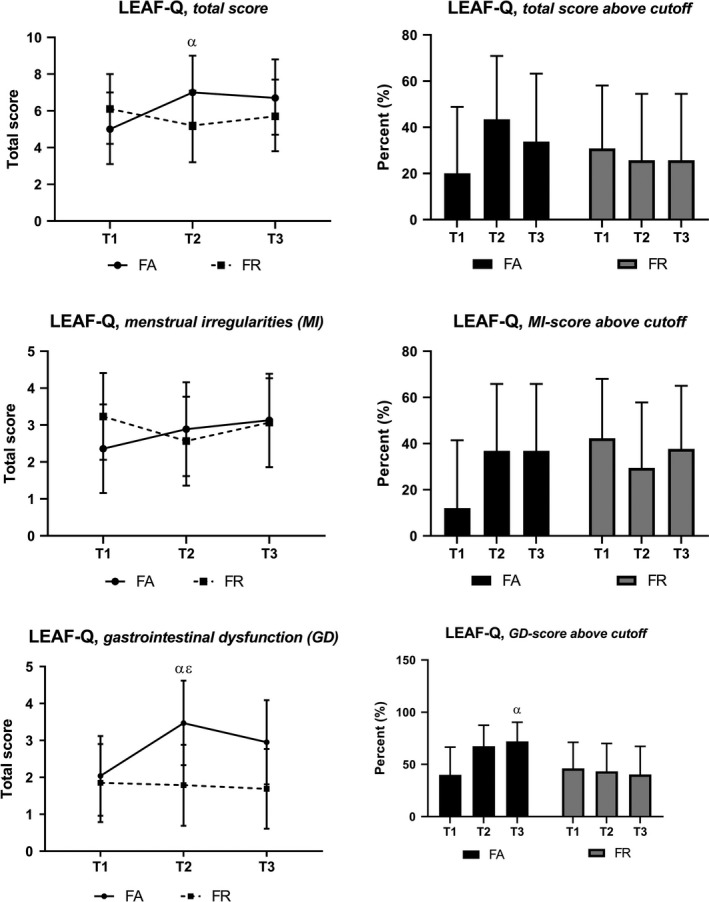
The scores from the LEAF‐Q (left side) and numbers above cutoff scores (right side). FA, fitness athletes; FR, female references; LEAF‐Q, the Low Energy Availability in Females Questionnaire; T1, baseline; T2, 2 weeks pre‐competition; T3, 1 month post‐competition; α, significant change from T1 in FA (*P* < .007); ε, significant between‐group difference (*P* = .006)

Results from LEAF‐Q are illustrated in Figure 2. We observed tendencies to statistically significantly increased numbers above the cutoff for LEAF‐MI in FA at T2 and T3 (*P* = .018), and a marginal between‐group difference in numbers above the cutoff for LEAF‐MI at T1 (*P* = .012).

Separate analysis according to use of HC found a significant increase in LEAF‐MI at T2 for FA not using any HC, with mean (99% CI) difference to T1 of 2.2 (0.04, 4.41) (*P* = .009). At T3, both FR and FA not using HC had significantly higher scores for LEAF‐MI compared to their corresponding affiliates using HC, with mean (99% CI) differences of 3.0 (0.73, 5.33) (*P* = .001) and 3.2 (0.49, 5.84) (*P* = .003), respectively. With regard to the LEAF subscale on gastrointestinal dysfunction (GD), only FA using HC increased LEAF‐GD at T2 by a mean (99% CI) of 2.5 (1.04, 3.96) (*P* < .001), resulting in a significantly increased LEAF‐total by 2.9 (0.49, 5.34) (*P* = .002).

### 3.4 Symptoms of depression (BDI‐1a)

At T2, the estimated mean (99% CI) BDI score increased from 2.6 (−0.3, 5.4) at T1 to 6.7 (3.7, 9.7) (*P* < .001) in FA, however, with no further difference to T1 at T3. There was no change by time in FR, with T1 score corresponding to 3.8 (1.0, 6.6). No between‐group differences occurred at any time. There were no findings of participants scoring above BDI cutoff at any time, other than one person from FR at T2 (BDI score of 24) and T3 (BDI score of 30).

### 3.5 Dietary intake

The results from dietary registration from T1 to T3 in each group are illustrated in Table 2. Additionally, the recommended levels for energy and nutrient intakes are presented in Table 2 as references. By comparison, carbohydrate intake in both groups is well below the suggested optimal range for physical activity and performance, while protein intake is in the upper range.

**Table 2:** Energy and nutrient intake from dietary registration in FA and FR, and with RDI as reference comparison

	RDI^a^	FA			FR			Between‐group differences (FR vs FA), ***Effect size (e)***, *P*‐value		
		T1	T2	T3	T1	T2	T3	T1	T2	T3
Energy, kcal		1783 _(1565, 2000)_	1502 _(1270, 1733)_ *e = 1,39* ***P* = .002**	2018 _(1774, 2263)_ *e = 0.61* ***P* = .01**	1832 _(1616, 2048)_	1955 _(1729, 2182)_	1900 _(1677, 2123)_	n*.s.*	455 _(130, 778)_ *e = 1.12* ***P* < .001**	n*.s.*
Energy, kcal kg LBM^−1^	>30^a^^,^ b	40 _(35, 45)_	33 _(28, 38)_ *e = 0.84* ***P* = .001**	43 _(37, 49)_ n*.s.*	42 _(37, 47)_	45 _(40, 50)_ n*.s.*	43 _(38, 48)_ n*.s.*	n*.s.*	13 _(5, 20)_ *e.=1,35* ***P* < .001**	n*.s.*
Carboh., gram		158.3 _(122.7, 193.8)_	116.9 _(79.1, 154.7)_ *e = 0.80* ***P* = .004**	185.3 _(145.5, 225.0)_	175.6 _(140.3, 210.9)_	195.4 _(158.5, 232.3)_	187.7 _(151.3, 224.1)_		78.5 _(25.6, 131.3)_ *e.=1.17* ***P* < .001**	
Carboh., g kg BW^−1^	5.0‐7.0^a^	2.6 _(2.0, 3.2)_	2.0 _(1.4, 2.7)_ n*.s.*	3.1 _(2.4, 3.7)_ n*.s.*	2.8 _(2.2, 3.4)_	3.0 _(2.4, 3.7)_ n*.s.*	2.9 _(2.3, 3.5)_ n*.s.*	n*.s.*	1.0 _(0.1, 1.9)_ *e = 0.87* ***P* = .003**	n*.s.*
Dietary fiber, g	25‐35^a^	25.7 _(19.9, 31.4)_	20.9 _(14.7, 27.1)_ n*.s.*	24.7 _(18.1, 31.3)_ n*.s.*	25.0 _(19.2, 30.7)_	25.6 _(19.5, 31.6)_ n*.s.*	21.2 _(15.3, 27.1)_ n*.s.*	n*.s.*	n*.s.*	n*.s.*
Protein, gram		160.7 _(141.4, 179.9)_	173.1 _(152.6, 193.7)_	150.1 _(128.4, 171.7)_	98.1 _(78.9, 117.3)_	110.0 _(89.9, 130.1)_	100.9 _(81.1, 120.7)_	−*62.5 _(−89.7, −35.4)_* *e = 2.08* ***P* < .001**	−*63.1 _(−91.8, −34.4)_* *e = 1.61* ***P* < .001**	−*49.2 _(−78.5, −19.8)_* *e = 1.19* ***P* < .001**
Protein, g kg BW^−1^	1.2‐2.0^a^	2.6 _(2.3, 2.9)_	3.0 _(2.7, 3.3)_ *e=−1.16* ***P* = .003**	2.5 _(2.1, 2.8)_ n*.s.*	1.6 _(1.2, 1.9)_	1.7 _(1.4, 2.1)_ n*.s.*	1.6 _(1.2, 1.9)_ n*.s.*	−1.0 _(−0.6, −1.5)_ *e=−1.92* ***P* < .001**	−1.3 _(−0.8, −1.8)_ *e=−1.96* ***P* < .001**	−0.9 _(−0,4, −1.4)_ *e=−1.33* ***P* < .001**
Fat, energy %	>20^a^	25.5 _(21.1, 29.9)_	20.1 _(15.4, 24.9)_ *e = 0.61* ***P* = .005**	28.3 _(23.2, 33.3)_ n*.s.*	36.5 _(32.1, 40.9)_	34.7 _(30.1, 39.4)_ n*.s.*	34.9 _(30.4, 39.5)_ n*.s.*	11.0 _(4.8, 17.2)_ *e = 1.30* ***P* < .001**	14.6 _(8.0, 21.3)_ *e = 1.78* ***P* < .001**	n*.s.*

Note

All values from dietary registrations are estimated means (99% CI), with any within‐group change from T1 presented consecutively, and with any between‐group differences presented in separate columns.

Abbreviations: 99% CI, 99% confidence interval; BW, body weight; e, effect size of Hedges g; FA, fitness competitor; FR, female references; g, gram; kcal, kilocalorie; LBM, lean body mass; LBM, lean body mass; n.s., not significant; RDI, recommended daily intake; T1, baseline; T2, competition; T3, post‐competition.

a ACSM position stand 28;

b The recommended lowest energy intake per kg LBM is after subtraction of exercise energy expenditure; however, the results presented are before any such subtraction.

### 3.6 RMR and HR

**Figure 3:**
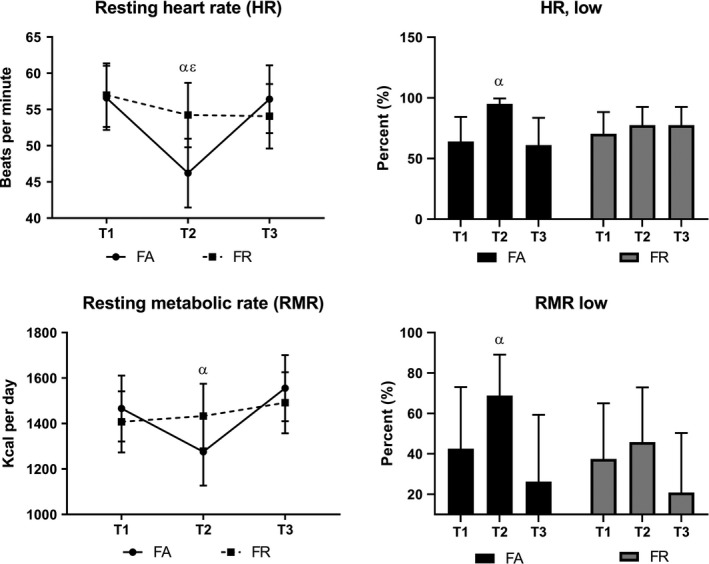
Changes in resting heart rate (HR) and resting metabolic rate (RMR)(left side), and numbers with clinically low HR and RMR (right side), during study period. FA, fitness athletes; FR, female references; T1, baseline; T2, 2 weeks pre‐competition; T3, 1 month post‐competition; α, significant change from T1 in FA (*P* < .009); ε, significant between‐group difference (*P* = .002)

Changes in RMR, resting HR, and numbers with clinically low RMR and HR are illustrated in Figure 3.

### 3.7 Body composition

**Figure 4:**
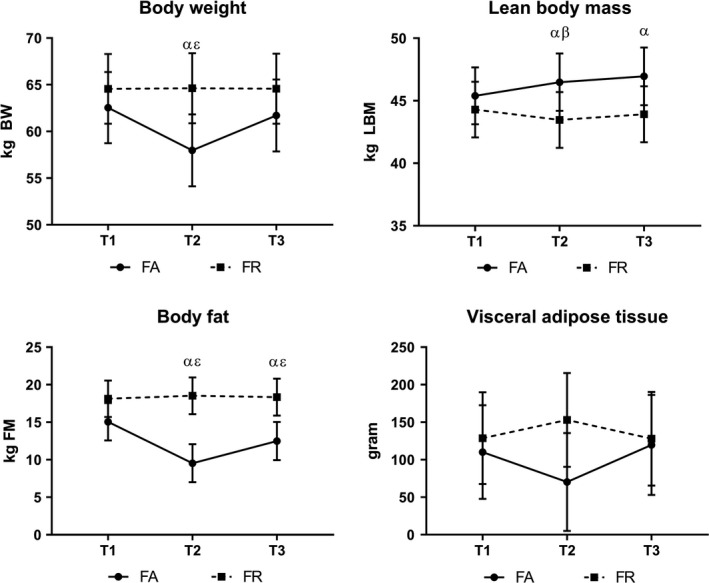
Changes in body weight and body composition during study period. BW, body weight; FA, fitness athletes; FM, fat mass; FR, female references; kg, kilogram; LBM, lean body mass; T1, baseline; T2, 2 weeks pre‐competition; T3, 1 month post‐competition; α, significantly different from T1 in FA (*P* < .002); β, significantly different from T1 in FR (*P* < .007); ε, significant between‐group difference (*P* < .006)

Changes in body composition during the study period (T1‐T3) are illustrated in Figure 4. In FA, mean (99% CI) %BF were 25% (22, 28) at T1, 17% (14, 20) at T2, and 21% (18, 24) at T3, with significant differences at T2 and T3 compared to T1 (*P* < .001). The corresponding findings for FR were 29% (26, 31), 30% (27, 32), and 29% (26, 32), with no significant within‐group change by time. There were between‐group differences for %BF at T2 and T3 (*P* < .001), and a marginal difference at T1 (*P* = .012). Marginal between‐group differences were also found for VAT at T2 (*P* = .018), and for LBM at T2 and T3 (*P* = .015) (Figure 4).

Only six (24%) FA and two (8%) FR had changes of detectable extent in spine BMD (of which five FA and one FR decreased spine BMD). The corresponding results for proximal femur BMD revealed no changes within limits of LSC in neither of the groups. Hence, there were no significant changes or between‐group differences in mean spine BMD, nor mean proximal femur BMD. At T1, estimated mean (99% CI) spine Z‐score was −0.04 (−0.52, 0.43) in FA and 0.43 (−0.03, 0.89) in FR, with no significant change by time within any of the groups. The mean (99% CI) femur Z‐score was 0.27 (−0.17, 0.72) in FA and 0.60 (0.16, 1.03) in FR at T1, with no significant change by time within any of the groups.

### 3.8 Subgroup analysis among the FA

Any differences at T2 between successful athletes and less successful athletes are illustrated in Table 3. Besides results presented in Table 3, there were no between‐group differences for LEAF‐Q scales, EDE‐Q or BMD at T2, nor were there any differences for the self‐reported history of, or current status of, EDs for these subgroups.

**Table 3:** Differences between successful athletes (FA placing among the top five athletes, FA‐5) and less successful athletes (FA placing below the top five athletes, FA‐0) in competitions (T2)

	FA‐5, n = 10	FA‐0, n = 11	*P*‐value, *effect size*
Age, years	29.1 (5.9)	27.7 (5.4)	.61
Numbers of previous competitions, n	2.2 (2.2)	0 (0)	**.01,** *e = 1.5*
T1‐T2 total BW lost, kg	−3.8 (3.8)	−5.4 (2.8)	.28
T1‐T2 change in LBM, kg	0.6 (1.6)	1.5 (1.6)	.19
Total fat mass at T2, kg	7.7 (1.3)	11.7 (2.7)	**.001.** *e = −1.9*
Total muscle mass at T2, kg	46.7 (2.2)	46.4 (5.6)	.87
%BF at T2, percent	14.2 (2.0)	20.1 (4.1)	**.001,** *e = −1.8*
VAT at T2, gram	28.1 (53.6)	105.7 (101.2)	**.01,** *e = −0.9*
RMR_m_/RMR_c_ at T2	0.79 (0.09)	0.88 (0.15)	.12
Low RMR at T2, n (%)	8 (80.0)	5 (50.0)	.07
HR at T2, BPM	43.8 (7.3)	48.1 (5.3)	.14
Low HR at T2, n (%)	10 (100)	10 (90.1)	.35
Energy intake at T2, kcal/ kg LBM	36.4 (5.2)	29.0 (6.7)	**.01,** *e = 1.2*
Carbohydrate intake at T2, g/kg BW	2.7 (1.0)	1.4 (0.9)	**.006,** *e = 1.4*
Protein intake at T2, g/kg BW	3.1 (0.5)	2.9 (0.7)	.21

Note

Results are presented as mean (SD) if not otherwise stated.

Abbreviations: %BF, body fat percentage; BPM, beats per minute; BW, body weight; e, effect size given as Hedges g; FA‐0, fitness athletes placing below top‐5; FA‐5, fitness athletes placing at top‐5; g, gram; HR, heart rate; LBM, lean body mass; RMR, resting metabolic rate; RMR_m_/RMR_c_, ratio of measured RMR to calculated RMR; T1, baseline; T2, competition time; VAT, visceral adipose tissue.

## 4 DISCUSSION

We aimed to evaluate the physical and mental health, and the occurrence of symptoms of relative energy deficiency in sport (RED‐s), in a sample of female fitness athletes (FA) during a dieting and post‐dieting period, and to study any differences to female references (FR). Additionally, we intended to study any differences between more and less successful FA. According to our first hypothesis, bone mineral density (BMD) was comparable between the two groups at baseline (T1), and fat mass was significantly lower in FA compared to FR. Contradicting our first hypothesis were comparable levels of lean body mass (LBM), symptoms of eating disorders (EDs), and depression between the two groups. In accordance with the second hypothesis, we found several symptoms of RED‐s in FA at competition time (T2). The low energy intake at T2 implies low energy availability, and the dieting in FA also resulted in a reduction in body weight (BW), fat mass, and resting heart rate (HR), and increased issues with gastrointestinal dysfunction (GD), all significantly different to FR. Additionally, at T2 there was a within‐group increase in FA in numbers with amenorrhea, in LEAF‐Q total score, a reduction in resting metabolic rate (RMR), and an increased number with clinically low RMR. Regarding our third hypothesis, some, but not all of the symptoms of RED‐s in FA resumed to T1 levels at post‐competition (T3). Specifically, numbers with amenorrhea and with GD‐scores above cutoff still significantly increased, and fat mass was below T1‐level and different to FR. Finally, supporting the last hypothesis was a finding of lower fat mass in more successful FA compared to less successful FA, but contradicting were the lack of difference in total LBM and outcomes related to RED‐s.

Our findings on high numbers with previous ED‐issues among FA correspond with previous reports,^1^, ^3^, ^4^, ^11^ suggesting this is a sport engaging females who endorse body appearance and disordered eating behavior. Nevertheless, of comparable concern is the relatively high occurrence of similar experiences among FR, which despite being a small group of recruited females seem to reflect the prevalence of ED in the general population.^39^ This finding may indicate that there are individuals within the sample of FR with personal motives and interests to the topic of this trial. Importantly though, this finding may interfere with our interpretation of the severity of issues with ED among FA.

While FR revealed small to no changes in LEAF‐Q scorings during this study, there were important fluctuations in FA. Menstrual irregularities and GD have previously been reported in athletes with LEA, and may partly be due to hormonal perturbations, but also a direct consequence of the lack of energy to support cell metabolism and function.^1^, ^6^ Our findings imply increased health and functional impairment with the extreme dieting in FA, and some with persistent effects beyond the dieting period. The increased number with amenorrhea in FA at T3 underlines this finding. Amenorrhea is previously reported in FA, with long‐term persistence despite normalization of BW and composition.^14^, ^15^, ^17^, ^20^ Present guidelines suggest BF% of 12% to be the lowest recommended level in females to avoid negative health effects.^6^ In the current study, the FA reached their lowest mean BF% of 17% at T2, while case studies of FA have reported levels of 9%‐11%^14^, ^15^, ^16^, ^17^ and cohort studies means of 13%‐18%.^20^, ^21^, ^22^ Importantly, in the previous case studies, menstrual disturbances were reported to occur early during the dieting,^14^, ^17^ suggesting such impairments may be a response to the LEA rather than a specific level of BF%.

The increase in BDI‐total score in FA at T2 indicates a change in mood, with increased symptoms of depression. However, the mean score was well below any clinical significance, and none was above the cutoff score indicating depression. Hence, the low energy intake may have deprived the FA of energy and vigor, comparable to what has been reported previously^1^, ^3^, ^16^, ^20^; still, the excitement over any successful dieting results and the competition coming up may have contradicted any overall severe change in mood.

In line with previous findings in these athletes, the diet‐induced changes in BW and body composition were temporary and returned quickly to baseline during the post‐competition period.^14^, ^17^, ^20^, ^21^ Noticeable, however, was the ongoing and permanent increase in LBM despite the energy restriction during the dieting period. One reasonable explanation to this increase may be a rather novice experience with resistance exercise, as FA were not different from FR in LBM or exercise history at T1. Still, previous publications report on comparable baseline values of LBM and increases in LBM while dieting, independent on whether athletes were described as novice^14^, ^20^ or experienced.^15^, ^16^, ^17^, ^20^ Hence, when complying with a structured resistance exercise program and a high‐protein diet, the stimuli for muscle growth most likely override the negative impact of negative energy balance, and at least in the short term, athletes may increase LBM. Nevertheless, the different responses in the recovery of body fat compartments from T2‐T3, with a faster restoration of VAT compared to total body fat, require some considerations. With no further follow‐up, it is hard to predict whether the VAT stabilizes, or continues to increase as a super‐compensation to the period with energy deprivation.^40^, ^41^ An additional pending question is whether repeated cycles of such diets impair optimal regulation of VAT.^40^, ^41^, ^42^, ^43^ With VAT more strongly associated with morbidity and mortality,^22^, ^44^ and with reports of increased difficulties in regulating BW after a career in aesthetic sports or after repeated weight cycling,^5^, ^40^, ^45^ worries on the long‐term metabolic health are reasonable. A recent finding on the upregulation of genes associated with adverse cardiovascular outcomes following BW regain in female physique athletes further emphasizes this concern.^22^

We hypothesized to find impairments in site‐specific BMD after dieting, but mean change in BMD was below the precision reliability of the DXA.^37^ Restrictive eating, menstrual disturbances, low BF%, and reduction in body weight have all been found to cause impairments in BMD.^7^, ^8^ Still, a period of more than 4‐6 months may be needed to detect any changes in BMD,^24^ which exceed the duration of the current study. Unfortunately, the design and resources available confined us from a longer follow‐up, and as such, a question remains regarding the effects from this sport on BMD.

In an attempt to find criteria for successful outcomes in this sport, we evaluated the most successful athletes toward the less successful ones. The most successful athletes were more experienced in competitions, consumed more carbohydrates and total energy, and were leaner at competition time, compared to the less successful ones. Low total fat mass is associated with low energy availability and high risk of several symptoms of RED‐s,^5^, ^6^, ^7^, ^23^ and the tendency of a clinically reduced RMR with the more successful FA in the current sample may indicate such a scenario. The findings from this subgroup analysis may highlight important practice for success in this sport, but do also bring up concern for health among the most successful athletes. Unfortunately, our results may have been tempered by the low number in each FA‐subgroup, and also constrained by a somewhat higher %BF in the current sample compared to previous reports in female FA.^14^, ^15^, ^16^, ^17^, ^20^

The FA did practice dieting recommendations^3^, ^5^, ^6^, ^9^ such as high meal frequency, high protein intake and moderately high‐fiber diets, and followed a moderate progress in BW changes, with a mean BW loss of 4.6 kg after ~12 weeks of dieting (ie, mean loss of BW of 0.59% per week). Nevertheless, while energy intake is increased post‐competition, restoring body weight and normalizing RMR and HR within a short timeframe, some hormonal and functional systems may seem to need more time to recover. This is of special concern considering the fact that some individuals prolong their dieting period for several weeks or months, or initiate several dieting periods per year. Hence, along with the acute impairments comes a risk of negative long‐term consequences,^6^, ^7^, ^8^ which specifically may be detrimental to young females with less robust hormonal systems.^6^, ^7^ The findings in the current study reveal female FA experience several symptoms of RED‐s during and after a dieting period.

### 4.1 Strengths and Limitations

The design of this study, including a reference group, using high‐quality and validated psychometric and physiological measures, and having a comparable or even higher number of participants to previous studies of similar athletic samples, adds credibility to the findings. Still, limiting the conclusions and interpretations of our findings are the short duration of follow‐up, a high number of the participants using HC (ie, depriving us of a number of participants in evaluation of hormonal effects from dieting), indications of having a selected sample of FR, and inadequate details on exercise during the FA dieting period depriving us of proper calculation on energy availability. Nevertheless, regarding the latter concern, an accurate calculation of energy consumption in resistance exercise is a matter of discussion due to its intermittent and partly anaerobic nature.^46^ With the mean energy intake in FA at T2 comparable to the suggested lowest appropriate energy availability,^29^ and considering the additional impact of a correction by energy consumption from exercise, there is a strong indication of LEA in FA. Finally, with no blood samples, there is no proper control of any use of illegal drugs. However, we included anonymous reports by questionnaires (none admitted using drugs), and one may assume the young athletes are of no interest to risk their career and penalties following any positive test.

## 5 CONCLUSION

Despite complying with the recommendations for athletes on BW loss, FA were not able to avoid impairment in health, indicative of LEA. Fortunately, the most negative outcomes from dieting were reversed post‐dieting, but an increased number with amenorrhea and continued presence of GD after dieting brings up concern for the long‐term health of athletes competing for several seasons. This concern does also apply to those adhering to the extreme modern, female body ideal, emphasizing a lean and toned muscular look.

## 6 PERSPECTIVE

Weight‐class and aesthetic sports involve periods with imposed BW loss, and the use of harmful weight control methods has repeatedly been reported.^5^, ^6^, ^7^ Many of the best‐practice recommendations on BW regulation, disclosed by experts in the field of physiology, dietetics, and sports,^5^, ^6^, ^9^, ^10^ are in fact practiced by fitness physique athletes.^11^, ^12^, ^13^, ^14^, ^15^, ^16^, ^17^ Albeit, our results reveal that negative health effects are unavoidable when emphasizing extreme leanness. Our findings underscore previous suggestions on the necessity of prioritizing periodization of BW regulation.^5^, ^6^ Complying with the modern athletic, female body ideal, may result in ongoing restrictive eating routines while practicing high and intensive exercise volumes.^4^ The current findings support these observations, as the FA were leaner than FR, consumed an energy‐restricted diet already before initiating the diet for competition participation, and required smaller body fat adjustments before competition than previously reported.^12^, ^16^, ^20^ Hence, there seems to be a need for explicit guidelines communicated more specifically, toward this sport and its devotees. As many of the athletes and coaches do not read scientific papers,^11^ there is a need to communicate this knowledge by other means.

## CONFLICT OF INTEREST

The authors report no conflict of interests.
